# Impact of income diversification on rural household food security in Ethiopia

**DOI:** 10.1080/20421338.2023.2220636

**Published:** 2023-07-03

**Authors:** Girma Gezimu Gebre, Aneteneh Ashebir, Tibebu Legesse

**Affiliations:** 1 Department of Agribusiness and Value Chain Management, Faculty of Environment, Gender, and Development Studies, College of Agriculture, Hawassa University, Ethiopia; 2 The Japan Society for the Promotion of Science (JSPS) Postdoctoral Research Fellowship Programme, Ritsumeikan University, Kyoto, Japan

**Keywords:** Ethiopia, food security, income, propensity score matching, rural household

## Abstract

Using primary data collected from 462 farm households, this paper aims to examine the impact of income diversification on rural household food security in Ethiopia. A propensity score matching model was employed to analyze the impact of participation in both agriculture and non-agriculture activities on household food security. The results indicate that age, education level, household size, number of contacts with extension agents, and numbers of livestock in tropical livestock units have a significant effect on household participation in both agriculture and non-agriculture sources of income generation. The propensity score matching result suggested that participation in both agriculture and non-agriculture would increase the rural household food security status by 10.6% to 19.5%, mainly due to a positive effect of additional sources of income generation from non-agriculture activities as witnessed in present and past studies. Therefore, to make considerable improvement on the food security situation, there is need to promote and scale-up on-farm, off-farm and non-farm income-generating activities in rural areas to diversify income sources so as to improve food security status of the rural households in Ethiopia.

## Introduction

Agriculture is the backbone of the Ethiopian economy. This particular sector determines the growth of all other sectors and consequently the whole national economy. It contributes over 79% of foreign earnings, 34% of the gross domestic product (GDP), accounts for over 80% of the labour force, and is the major source of raw materials and capital for investment and market (Ministry of Agriculture (MOA) 2019). However, just 5% of land is irrigated and crop yields from small farms are below regional averages in Ethiopia. Market linkages are weak, and the use of improved seeds, fertilizers and pesticides remains limited (USAID 2022). Moreover, decrease in cultivable land size (IFAD 2011); progressive increase in population and frequent drought (Gebre and Rahut 2021); global COVID-19 pandemic (FAO 2020), and the most recently, war in its northern regions significantly damaged the growth of agricultural sector in Ethiopia. As a consequence, the present capacity of agriculture to achieve food and livelihood security has enormously declined. Ethiopia has become one of the most food insecure countries in sub-Saharan Africa (Gebre 2012; FAO et al. 2018; FAO 2020; Gebre and Rahut 2021).

Food security is an important matter of concern for countries across the globe. According to the level of focus (i.e., global, regional, national, community, household and individual), the term food security has different aspects. The importance of assuring food security at household-level received more attention of national governments as well as international communities in the last few decades (Zoungrana 2022). In 1996, the World Food Summit stated that ‘*when household food security exists, all people in a household have access to sufficient, safe, nutritious food at all times to maintain their active and healthy life*’ (FAO 1996). This definition integrates four dimensions of food security include: access to food, availability of food, the biological utilization of food, and the stability in all times (Zoungrana 2022).

The situation of food security in developing countries has become terrible. An analysis by (FAO et al. 2019) indicated that, in 2018, about 272 million undernourished people lived across countries in sub-Saharan Africa, accounts more than 35% of world undernourished people in 2018. From 272 million undernourished people, about 65% lived in East, followed by southern Africa (54%), West Africa (48%), and North Africa (30%) (FAO et al. 2019). This implies that most of the East African population does not have regular access to nutritious and sufficient food for a healthy and productive life (Gebre and Rahut 2021; Gebre et al. 2023a; Gebre et al. 2023b). The problem of access to adequate and sufficient food for the population is a major concern in Ethiopia.

Smallholders, who constitute 98% of Ethiopian farmers (Dufera 2018), are pursuing various adaptation strategies in attempts to develop resilience that may help them overcome challenges and reduce their level of vulnerability to food insecurity. One of the adopted strategies is income source diversification, which is defined as increasing smallholder farmers or household income sources rather than farming activities like crop production and livestock rearing (Hengsdijk et al. 2007). According to Adem et al. (2018), diversification is the process by which households widen their income base by adopting new economic activities. A study by Michlera and Josephson (2017) in Ethiopia, suggests that families who grow a diverse set of crops are less likely to be poor as compared with households specializing in their crop production. Studies by Nagler and Naudé (2013) and Yizengaw (2014) in Ethiopia, Babatunde and Qaim (2010) in Nigeria, and Dev, Sultana, and Hossain (2016) in Bangladesh noted that income diversification provides additional income that relaxes the financial constraint on households, either to purchase from market (Yizengaw 2014; Dev, Sultana, and Hossain 2016) or increasing their own production by easing capital constraints on the households (Babatunde and Qaim 2010). Subsequently, households spend more on their basic needs including food, clothing, education, and healthcare. Thus, multiple sources of income with reliable amounts are essential to ensuring food for households. Babatunde and Qaim (2010) indicated that the prevalence of child stunting and underweight was lower among farmers with off-farm incomes as compared with families without off-farm incomes in Nigeria. Studies by Endalew, Muche, and Tadesse (2015), Yenesew (2015), and Abduselam (2017) noted that diversification of non-farm^1^ and off-farm^2^ activities (such as off-farm employment, petty trade, and selling charcoal and firewood) was used as a coping strategy during severe food insecurity among rural households in Ethiopia.

Income diversification was associated with food security in Burkina Faso (Reardon, Delgado, and Matlon 1992; Zoungrana 2022); Nigeria (Ibekwe et al. 2010; Dedehouanou and McPeak 2020), Bangladesh (Dev, Sultana, and Hossain 2016), Kenya (Nyariki, Wiggins, and Imungi 2002; Olale and Henson 2012), and Ethiopia (Birhanu et al. 2010; Nagler and Naudé 2013; Yizengaw 2014; Adem et al. 2018; Etea et al. 2019; Kidane and Zegeye 2020; Gebrtetsadik 2022). For rural households, participation in off-farm activities is crucial to reduce their food insecure status in Ethiopia. Kilic et al. (2009) noted that most poor households’ income from the farm is not enough for the whole year’s consumption in Ethiopia, and they use off-farm income in the crucial hungry period between food stores running out and the next harvest. Therefore, off-farm income can be used as a mechanism to stabilize the household income and reduces early harvest consumption or distress selling at early harvest time in Ethiopia (Adem etal. 2018).

Food insecurity in Ethiopia is derived primarily from dependence on undiversified livelihoods based on low-output rain-fed agriculture (Devereux 2000). However, studies linking household income diversification to food security in Ethiopia are limited. Most of the available studies (e.g., Mathewos 2013; Gecho 2017; Yenesew, Eric, and Fekadu 2015; Abduselam 2017; Wondimagegnhu, Huluka, and Nischalke 2019) relied only on factors influencing decision to farm diversification and determinants of livelihood diversification. Therefore, this paper contributes to the literature on income diversification and food security by examining the impact of income diversification on rural household food security in Ethiopia. Study answers the following two research questions: (1) Are there any significant differences in food security status between participants in only agriculture and in both agriculture and non-agriculture activities?^3^ (2) What is the average treatment effect of participation in both agriculture and non-agriculture activities on smallholder household food security? It is hypothesized that participant in both agriculture and non-agriculture activities would exhibit a much higher food security status in comparison to the control group (participant only in agriculture) due to the positive effect of additional income sources from non-agriculture activities as witnessed in various empirical studies.

## Materials and methods

### Study area, data and survey design

The study is based on the primary data collected through the Stress Tolerant Maize for Africa (STMA) project in Ethiopia in December 2019. The identification and selection process of study areas and respondent households were designed by researchers from the International Wheat and Maize Research Centre (CIMMYT) in collaboration with regional and woreda (district) level Ministry of Agriculture personnel in Ethiopia. Accordingly, the respondents and target districts were selected from three regions: Amhara (Guangua, Bure and Jabi Tehnan districts), Oromia (Zeway Dugda, Adama, Adami Tulu and Jido Kombolcha, Omonada, Arsi Negele, Shala, Shashemene, and Siraro districts), and the South Nation, Nationalities, and People region (Arba Minch Zuria, Mirab Abaya, Mareqo, Misraq Badawacho and Boloso Sore districts). The identification and selection process of study districts were based on their potential for major crop production in the country. The major producing crops in these study districts include maize, teff, haricot bean, wheat, sorghum, pepper, and finger millet.

Respondents, districts (woreda) and sub-districts (kebele) were selected for the survey by means of a multi-stage sampling procedure that involved a combination of purposive and random sampling. The major crop producing districts and sub-districts (kebeles) were purposively identified based on their current production potential and status. Proportional to size, the random sampling procedure was used to select 1–2 kebele per district, where 18–20 farm households per kebele were selected from a complete household list provided by local authorities. A total of 516 households were interviewed in 2019. A semi-structured questionnaire was designed and tested to capture a range of information related to household demographic, socioeconomic, and agronomic features and food security. The questionnaire also captured some individual household characteristics, as well as institutional arrangements besetting households in farm management. Well trained enumerators administered the questionnaire under the close supervision of researchers from CIMMYT. Before the analysis, a thorough data cleaning was performed. The analysis was carried out based on balanced data obtained from 462 households, using the STATA version 17.

### Methods of data analysis

The collected data were analyzed using descriptive and econometric models. Descriptive statistics such as frequency, percentage, and mean were used. Chi-square and t-test were used to examine the difference between two groups in terms of categorical and continuous variables, respectively. The propensity score matching (PSM) model was employed to estimate the impact of diversifying income sources on household food security in rural Ethiopia. The reason for the application of this model was a high potential in reducing bias through matching those who participated in both agriculture and non-agriculture activities as sources of their household income with those who participated in agriculture as their only source of income. PSM estimation involves the identification of the probability of participation in both agriculture and non-agriculture sources of income generation. When estimating the propensity score, two choices must be be made. The first is estimating the probability of participation in both agriculture and non-agriculture sources of income generation, and the second is about the variables to be included in that model (Caliendo and Kopeinig 2005). For binary dependent variables, the models that are used to estimate the probability of participation against non-participation households are logit or probit models. However, the choice between the two is not problematic as they provide the same result (Gujarati and Porter 2009). Therefore, in this paper the logit model was applied to determine the propensity score of the households.

The expected treatment effect for the treated population is of primary significance in applying PSM method and given as^4^

(1) $$\text{ATT}=E(\Delta |D=1)\;\;=E(Y_{1}|x,\;D=1)-E(Y_{0}|x,\;D=1)$$

where ATT is the average treatment effect for the treated, $Y_{1}$ denotes the value of the outcome for participants in both agriculture and non-agriculture income-generating activities and $Y_{0}$ is the value of the same variable x for those who participates only in agriculture activities. As noted above, the major problem is that we do not observe $E(Y_{0}|x,\;D=1)$, which is counterfactual. Although the difference $\text{ATT}=(Y_{1}|x,\;D=1)-E(Y_{0}|x,\;D=0)$ can be estimated, it is potentially a biased estimator.

In the absence of experimental data, the PSM can be employed to account for this sample selection bias (Dehejia and Wahba 2002). The PSM is defined as the conditional probability that a farm household participates in both agriculture and non-agriculture income-generating activities given pre-participation (participate only in agriculture) characteristics (Rosenbaum and Rubin 1983). To create the condition of a randomized experiment, the PSM employs the unconfoundedness assumption, also known as conditional independence assumption, which implies that once Z is controlled for, participation in both agriculture and non-agriculture income-generating activities is random and uncorrelated with the outcome variables (food security in our case). In short, the outcomes are independent of treatment. The PSM can be expressed as:

(2) P(Z)=Pr{D=1∣Z}=E{D∣Z}

where D = (Abara and Singh 1993) is the indicator for participation in both agriculture and non-agriculture income-generating activities and Z is the vector of pre-participation (participation only in agriculture) characteristics. The conditional distribution of Z, given P(Z) is similar in both groups, namely those who participate in both agriculture and non-agriculture income-generating activities and those who participate only in agriculture-related activities. After estimating the propensity scores, the average treatment effect for the treated (ATT) can then be estimated as:

(3) $$\text{ATT}=E\{Y_{1}-Y_{0}|x,\;D=1\}\;\;=E\{E\{Y_{1}-Y_{0}|x,\;D=1,\;p(Z)\}\}\;\;=E\{E\{Y_{1}|x,\;D=1,\;p(Z)\}\;\;-E\{Y_{0}|x,\;D=0,\;p(Z)\}|x,\;\;D=0\}$$

Several techniques have been developed to match participation in both agriculture and non-agriculture activities and participation only in agriculture activities of similar propensity scores. In the case of PSM, the most important variable of interest is the average treatment effect for the treated (ATT) (Ali and Erenstein 2017). ATT is the difference in the outcome of farm households having engaged in both agriculture and non-agriculture activities as income sources and similar farm households engaged only in agriculture activities. For estimation in the current paper, four different matching algorithms, i.e., nearest neighbour, radius, kernel-based, and stratified matching are employed. A matching estimator that bears low pseudo R^2^, results in large matched samples and insignificant explanatory variables after matching should be chosen (Dehejia and Wahba 2002).

In the current paper, we use PSM to estimate the impact of income diversification activities on household food security. The food security status or cut-off point was calculated using the Household Food Insecurity Access Prevalence (HFIAP) indicators that were developed by the United States Agency for International Development (USAID) Food and Nutrition Technical Assistant (FANTA) project. For each household, the Household Food Insecurity Access (HFIA) category variable was calculated using the assigned codes of the degree of food security states into which it fell. Accordingly, four categories of food security states were created sequentially, (1 = food secure, 2 = mildly food insecure, 3 = moderately food insecure, and 4 = severely food insecure), to ensure that respondent households were classified according to their most severe response. Each category was calculated by dividing the number of households in one category by the total number of households in the four categories. Due to the small sample size, we merged three food insecure statuses (mildly, moderately, and severely) into ‘food insecure’ and the rest into ‘food secure’ categories. Thus, the dependent variable (outcome variable) was binary, with ‘one’ assigned to the food secure household category and ‘zero’ to the food insecure household category.^5^ The impact was estimated on participation in both agriculture and non-agriculture activities by farm households to generate income. For instance, if one farm household participates in both agriculture and non-agriculture activities, how great is the impact on that household’s food security status? A description of the variables included in the model estimation and hypothesis are presented in Table 1.

## Results and discussion

### Descriptive results

Tables 2 and 3 present summary statistics of categorical and continuous variables included in the model. Out of the surveyed households, 33.12% participated in both agriculture and non-agriculture activities, while 66.88% participated only in agriculture as a source of their income.

The mean age of participants in both agriculture and non-agriculture sources of income generation was found to be 49.16 years and 48.03 years of those involved only in agriculture. The mean years of education for participants in both agriculture and non-agriculture sources of income generation as well as those with only agriculture income source were 4.15 and 3.01, respectively. This shows that most of the households had education up to primary school level. A difference in mean education level between participants in both agriculture and non-agriculture sources of income generation and only agriculture income source was statistically significant at a 1% level. The mean education level of spouses of participants in both agriculture and non-agriculture sources of income generation and only agriculture income source was 2.06 and 1.57, respectively. The difference is statistically significant at the 10% significance level.

It is evident from the results that participants in both agriculture and non-agriculture sources of income generation have larger family sizes than only agriculture sources of income generation participants. The average family size was 7.06 and 6.41 in both agriculture and non-agriculture sources of income generation and only agriculture sources of income generation, respectively. The difference in family size between the two groups was significant at a 1% significance level.

The average farm size of participants in both agriculture and non-agriculture sources of income generation was 1.94 hectares, while that of participants with only agriculture sources of income was 2.018 hectares. The results reveal that participants in both agriculture and non-agriculture sources of income generation have higher numbers of contacts with extension agents than those with only agriculture sources of income generation. The average number of contacts with extension agents per month was 3.86 and 3.29 between participants in both agriculture and non-agriculture sources of income generation and only agriculture sources of income generation, respectively. The difference in the number of contacts with extension agents between the two groups is significant at a 5% significance level. The mean distance to the main market was 8.1 km for participants in both agriculture and non-agriculture sources of income generation while it was 7.79 km for participants in only agriculture sources of income generation. The mean distance from the agriculture office for participants in both agriculture and non-agriculture sources of income generation and only agriculture sources of income generation was 2.66 and 2.73 km, respectively. Furthermore, the average livestock ownership of households measured in tropical livestock units was 4.63 for participants in both agriculture and non-agriculture sources of income generation while it was 3.00 for participants in only agriculture sources of income generation. The difference in livestock ownership of households in tropical livestock units between the two groups is significant at a 5% significance level.

Out of the surveyed households, 91.56% were male-headed while, 8.44% were female-headed. The proportion of male-headed households participants in both agriculture and non-agriculture sources of income generation and participants in only agriculture sources of income generation was 90.85% and 91.91%, respectively. In terms of membership of cooperatives, out of the surveyed households, 42.1% were members in cooperatives. Out of participants with both agriculture and non-agriculture sources of income generation, 43.14% were members of a cooperative while 56.86% were non-members. Similarly, 41.75% of participants in only agriculture sources of income generation were members of cooperatives while 58.25% were non-members. From participants in both agriculture and non-agriculture sources of income generation, 21.57% participated in demonstration visits while 19.42% who participated in only agriculture sources of income took part in demonstration visits.

### Determinants of diversifying income sources

This sub-section presents the result of the logistic regression model, which was used to estimate the propensity score for matching the participant households in both agriculture and non-agriculture sources of income generation and participants in only agriculture sources of income generation. As shown in Table 4, the model sufficiently fitted the data at less than a 1% significance level (LR chi^2^ (13) = 155.27; Prob > chi^2^ = 0.0004). The pseudo-R^2^ value is 0.0601, which is fairly low, indicating that participants in both agriculture and non-agriculture sources of income generation do not have much distinctiveness in overall characteristics, making the matching between participants with both agriculture and non-agriculture sources of income generation as well as only agriculture income source easier. Thirteen (13) explanatory variables were used to estimate the determinants of household participation in both agriculture and non-agriculture sources of income-generation activities. The result of the logistic regression shows that age of households, education level of the household head, household size, number of contacts with extension agents and number of livestock in tropical livestock units were the factors that significantly influence smallholder household participation in both agriculture and non-agriculture income-generating activities compared with participation in only agriculture (on-farm) income-generating activities.

The results indicate that there is a positive association between the age of the household head and the probability that a household will participate in both agriculture and non-agriculture sources of income generation. As the age of a household head increases by one year, increase the chance of being to participate in both agriculture and non-agriculture income-generating activities by 2.3% at less than 1% significance level. The probable reason is that age of the household helps to participate in additional income source through experience. This result is consistent with a study by Ampaw, Nketiah-Amponsah, and Senadza (2017) in Ghana, who found a positive relationship between the age of farmers and the probability of their participation in non-farm and off-farm activities.

Education level of the household head had a positive and significant effect on the probability of participation in both in agriculture and non-agriculture sources of income generation at a less than 1% significance level. The results of the odds ratio, which indicates the strength of the association, suggest that increase in education level by one year could increase the probability of participation in both agriculture and non-agriculture sources of income generation by 12.6%, *ceteris paribus*. This could be because education improves human capital and opens up additional job opportunities for farm households. This result is in line with the findings of Seng (2015) in Cambodia, Adem et al. (2018) in Ethiopia, and Anang, Nkrumah-ennin, and Nyaaba (2020) in Ghana, who suggested that more educated farmers are more likely to work off-farm sources of income generation compared with less educated farmers.

The number of household members has a positive influence on participation in both agriculture and non-agriculture sources of income generation. The odds ratio results suggest that an additional family member in the household would increase their participation in both agriculture and non-agriculture activities by 12.2%. This possibly accounts for the fact that households with more members would have abundant labour and are more likely to participate in both agriculture and non-agriculture activities. This result is consistent with the findings of Ahmed and Melesse (2018) and Anang, Nkrumah-Ennin, and Nyaaba (2020) who found that family size increases the probability of diversifying in non-farm income activities in Ethiopia and Ghana, respectively.

The study also found a positive association between frequency of contacts with extension agents and the probability of being involved in both agriculture and non-agriculture income-generating activities. The odds ratio indicates that the number of contacts with extension agents increases the probability of participation in both agriculture and non-agriculture sources of income generation by 8.7% at a statistically 1% significance level. This result could be because households with more access to extension services could have increased opportunities to be involved in awareness creation training and access to information about income diversification strategies. This result is in line with the findings by Amsalu et al. (2013), Agyeman, Asuming-Brempong, and Onumah (2014) and Dagunga et al. (2018).

The number of livestock in tropical livestock units was found to have a positive impact on participation in both agriculture and non-agriculture income-generating activities at a 5% level of significance. For one unit increase in the value of livestock, the probability of participation in both agriculture and non-agriculture sources of income generation would increase by 12.2%. This result is in line with the findings by Ampaw, Nketiah-Amponsah, and Senadza (2017) and Yizengaw (2014) who found a positive relationship between the number of livestock and the probability of participation in non-farm and off-farm activities.

### The impact of participation in both agriculture and non-agriculture sources of income generation on households’ food security

After estimating the logit model, we predicted the propensity scores and the common support region. The overall estimated propensity score was between 0.067 and 0.820, as depicted in Table 5. Among the treated (participate in both agriculture and non-agriculture) households, the score lies between 0.123 and 0.820 but for control (participate in only agriculture) households, it lies between 0.067and 0.747. After estimation of propensity score checking, the common support condition was the main task to process the next step. This indicates that according to minima and maxima approaches, the region of common support would lie between 0.123 and 0.747. Therefore, the propensity score approached less than 0.123 and the propensity score greater than 0.747 for control households and treated households, respectively, failed to lie in the common support region.

Accordingly, as shown in Table 6, only two observations were ignored from analysis from the treated group. Furthermore, the overall average propensity score among the households was 0.331 implying that the average probability to participate in both agriculture and non-agriculture sources of income generation among sampled households was about 33%.

**Figure 1:**
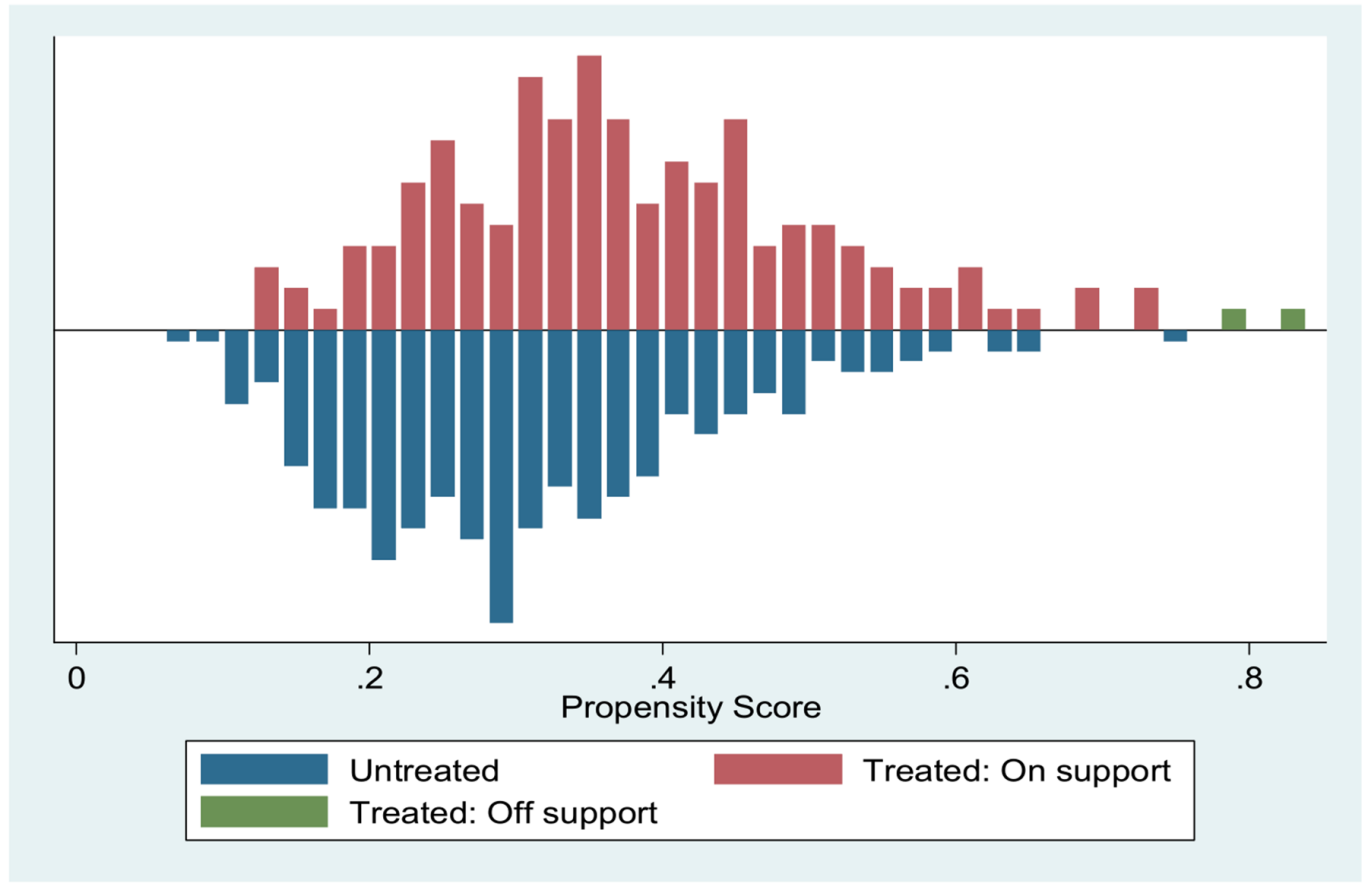
Distribution of propensity scores.

Figure 1 shows the visual presentation of the density distribution of the estimated propensity scores for treated households and control households. It shows the number of households on support and those off support. The figure reveals substantial overlap in the distribution of the propensity scores of both treated households and control households. This confirms that matching requirements were satisfied for computing treatment effects.

Table 7 shows the results of the Average Treatment Effects on Treated (ATT) of the outcome variable using propensity score matching techniques. The average treatment effect for the treated (ATT) was estimated using four algorithms, nearest-neighbour, kernel, stratification matching, and radius matching. The results for the estimated treatment effect on treated households show that participation in both agriculture and non-agriculture sources of income generation would increase rural household food security status by 10.6% to 19.5%.

The estimated impacts were 0.106, 0.195, 0.146 and 0.158 for nearest-neighbour, kernel, stratification matching and radius matching, respectively. As shown in Table 7, kernel matching gives highest results of a 0.195 increase in the food security status due to participation in both agriculture and non-agriculture sources of income generation. Thus, the results indicate that participation in both agriculture and non-agriculture sources of income generation would increase the probability of being food secure by 10.6% to 19.5%. These results validate a prior hypothesis of this study which predicts participation in both agriculture and non-agriculture activities would exhibit a much higher food security status in comparison to participation only in agriculture. The present study results are in line with prior studies by Endiris, Brehanie, and Ayalew (2021) and Yenesew and Masresha (2019) in Ethiopia. Moreover, a study by Osarfo, Senadza, and Nketiah-Amponsah (2016) in Ghana reported a similar finding that non-farm employment has a statistically significant positive effect on the income of households as well as their food security status. A study by Chirwa et al. (2017) in Malawi also found that income diversification has a positive impact on household food security.

## Conclusion

Using data collected from 462 households, this study applied a propensity score matching procedure to explore the impact of income diversification on household food security among farmers in rural Ethiopia. The result of the logit model indicated that age, education level, household size, number of contacts with extension agents, and numbers of livestock in tropical livestock units have a significant effect on households’ participation in both agriculture and non-agriculture sources of income generation. The propensity score matching results show that participation in both agriculture and non-agriculture sources of income generation would increase rural household food security status by 10.6% to 19.5%. Hence, the study concludes that households who actually participate in both agriculture and non-agriculture sources of income generation would have, on average, a higher probability of being food secure than farm households who participate in only agriculture as a source of their income. Therefore, to make considerable improvements in the food security situation, there is need to focus on promoting income diversification strategies such as participation in non-farm and off-farm activities in addition to participation in agricultural activities.

## Figures and Tables

**Table 1:** Description of the variables and hypothesis.

Explanatory variables	Variable type	Expected sign
Sex of the household head (Male = 1, Female = 0)	Dummy	−ve
Age of the household head	Continuous	−ve
Education level of the household head (year of formal schooling)	Continuous	+ve
Age of spouse	Continuous	−ve
Education level of spouse in years	Continuous	+ve
Participation in agriculture demonstration visit (1 = yes, 0, otherwise)	Dummy	+ve
Household size	Continuous	+ve
Size of the household landholding in hectare	Continuous	+ve
Number of contacts with extension agent	Continuous	+ve
Membership of agriculture input supply cooperatives (1 = yes, 0, otherwise)	Dummy	+ve
Distance to main market in km	Continuous	−ve
Distance to agriculture office in km	Continuous	−ve
Number of livestock in Tropical Livestock Unit (TLU)	Continuous	+ve

**Table 2:** Summary statistics of continuous variables included in model estimation.

	Total		Both Agri and non-agri		Agri-only		
Variable description	Mean	St. dev.	Mean	St. dev.	Mean	St. dev.	T-test
Age of the household head	48.40	12.67	49.16	13.56	48.03	12.21	−0.90
Education level of the household head	3.38	3.79	4.15	4.31	3.01	3.45	−3.06***
Age of spouse	39.13	9.64	38.35	8.50	39.52	10.15	1.22
Education level of spouse	1.73	2.82	2.06	3.05	1.57	2.69	−1.759*
Household size	6.84	2.36	7.06	2.47	6.41	2.03	2.788***
Land size of the household in hectare	1.99	1.08	1.94	1.06	2.02	1.10	0.689
Number of contacts with extension agents	3.48	2.73	3.86	2.36	3.29	2.88	−2.11**
Distance to main market in km	7.89	5.56	8.10	5.74	7.79	5.47	−0.56
Distance to agriculture office in km	2.71	4.65	2.67	3.19	2.73	5.23	0.156
Number of livestock in TLU	3.53	6.80	4.63	6.92	3.00	5.15	2.795**

***Source:*** Survey result; N (Pooled) = 462; N (Agriculture and non-agriculture sources of income generation) = 153; and N (Agriculture only) = 309 ***, ** and * Significant at 1%, 5 and 10 probability levels respectively.

**Table 3:** Summary statistics of discrete variables included in model estimation.

Parameter	Category	Total HHs		Both Agri and non- Agri		Agri-only		*χ* ^2^
		N = 462	%age	N=153	%age	N = 309	%age	
Sex of the household head	Male	423	91.56	139	90.85	284	91.91	0.700
	Female	39	8.44	14	9.15	25	8.09	
Participation in demonstration visit	Yes	93	20.13	33	21.57	60	19.42	0.587
	No	369	79.87	120	78.43	249	80.58	
Membership in cooperative	Yes	195	42.21	66	43.14	129	41.75	0.776
	No	267	57.79	87	56.86	180	58.25	

***Source***: Survey result

**Table 4:** Estimation of propensity score through logit regression.

Head agriculture plus other income (livelihood) sources	Coef.	Std. Err.	*P* > *z*
Sex of the household head	−0.417	0.386	0.279
Age of the household head	0.023	0.009	0.009***
Education level of the household head	0.126	0.031	0.000***
Age of spouse	−0.005	0.012	0.657
Education level of spouse	0.061	0.039	0.121
Participation in agriculture demonstration visit	0.159	0.256	0.534
Household size	0.122	0.046	0.009***
Size of the household landholding in hectare	−0.035	0.097	0.717
Number of contacts with extension agent	0.087	0.038	0.022**
Member in agriculture input supply cooperatives	0.081	0.214	0.702
Distance to main market	0.003	0.018	0.863
Distance to agriculture office in km	0.071	0.221	0.310
Number of livestock in TLU	0.121	0.106	0.005*
_cons	−1.348	0.821	0.101
Number of obs = 462			
Log likelihood = −275.73896			
LR chi2(13)= 155.27			
Prob > chi2 = 0.0004			
Pseudo R2 = 0.0601			

***, ** and * significance at 1%, 5% and 10% probability levels, respectively.

**Table 5:** Distribution of estimated propensity scores.

Categories	Obs	Mean	Std. dev.	Min	Max
Total households	462	0.331	0.129	0.067	0.82
Treated households	153	0.381	0.135	0.123	0.820
Control households	309	0.306	0.119	0.067	0.747

**Table 6:** Common support and treatment

Treatment assignment	Off support	On support	Total
Control households	0	309	309
Treated households	2	151	153
Total households	2	460	462

**Table 7:** Impact of diversifying sources of income generation on household food security.

Outcome indicator	Matching algorithms	Sample	Treated	Controls	Difference	S.E	t-stat
Food Security	Nearest neighbour matching	Unmatched	0.654	0.576	0.078	0.048	1.60
		ATT	0.656	0.550	0.106	0.050	2.10***
	Kernel matching	Unmatched	0.678	0.625	0.053	0.052	1.92
		ATT	0.721	0.526	0.195	0.092	3.32***
	Stratification matching	Unmatched	0.642	0.611	0.031	0.009	1.42
		ATT	0.692	0.546	0.146	0.023	2.47***
	Radius matching	Unmatched	0.472	0.411	0.061	0.044	1.73
		ATT	0.588	0.430	0.158	0.086	2.11***

***, ** and * significant at 1%, 5% and 10% probability levels, respectively.

## Footnotes

1. Non-farm income generated from activities outside of agricultural market systems. Non-farm sectors include construction, health care, hospitality, education, mining, and tourism.

2. Off-farm income encompasses all agriculture-related activities that occur beyond the farm. Off-farm income includes the ‘middle’ and ‘end’ of the process, as agricultural goods leave the farm to ultimately reach the consumer. Examples of off-farm income and enterprises include extension services, processing, packaging, storage, transportation and distribution, and retail sale.

3. This study defined a participant in agriculture as a household who engaged in on-farm activities and whose income was generated from only on-farm sources. A participant in non-agriculture is defined as a household who engaged in either or both non-farm and off-farm activities and whose income was generated from either or a combination of these two types of activities.

4. Expected treatment effect on the treated is the difference between the outcome of treated and the outcome of the treated observations if they had not been treated.

5. Regarding the food security status of the surveyed households, about 60% were in the food secure category, while the remaining 40% were in the food insecure category. In the food insecure category (out of the 40%), about 11% were mildly food secure, while the rest, 22% and 7%, were moderately and severely food insecure, respectively.
